# Distinct sets of locomotor modules control the speed and modes of human locomotion

**DOI:** 10.1038/srep36275

**Published:** 2016-11-02

**Authors:** Hikaru Yokoyama, Tetsuya Ogawa, Noritaka Kawashima, Masahiro Shinya, Kimitaka Nakazawa

**Affiliations:** 1 Department of Life Sciences, Graduate School of Arts and Sciences, The University of Tokyo, 3-8-1 Komaba, Meguro, Tokyo 153-8902, Japan; 2 Research Fellow of the Japan Society for the Promotion of Science, Tokyo, Japan; 3 Department of Rehabilitation for the Movement Functions, Research Institute of National Rehabilitation Center for the Disabled, 4-1 Namiki, Tokorozawa, Saitama 359-8555, Japan

## Abstract

Although recent vertebrate studies have revealed that different spinal networks are recruited in locomotor mode- and speed-dependent manners, it is unknown whether humans share similar neural mechanisms. Here, we tested whether speed- and mode-dependence in the recruitment of human locomotor networks exists or not by statistically extracting locomotor networks. From electromyographic activity during walking and running over a wide speed range, locomotor modules generating basic patterns of muscle activities were extracted using non-negative matrix factorization. The results showed that the number of modules changed depending on the modes and speeds. Different combinations of modules were extracted during walking and running, and at different speeds even during the same locomotor mode. These results strongly suggest that, in humans, different spinal locomotor networks are recruited while walking and running, and even in the same locomotor mode different networks are probably recruited at different speeds.

In locomotion, both animals and humans use several different locomotor modes to meet the demand for different movement speeds. In animals, for example, larval zebrafish exhibit both “slow swim” and “burst swim” modes by modifying their movement frequencies and kinematics^1^. Locomotion modes in horses have more variety, i.e., walk, trot, pace, canter and gallop, on the basis of different patterns of ground contact^2^.

Regarding the neural mechanisms underlying locomotion, experimental studies using animal models in the last few decades have provided explicit evidence for the existence of spinal central pattern generators (CPGs), and have revealed that these CPGs consist of spinal interneuron networks that are responsible for generating locomotor rhythms and patterns^3^,^4^. In humans, recent studies on spinal cord-injured (SCI) patients^5^,^6^,^7^,^8^ and on healthy participants^9^ have provided indirect evidence of the existence of CPGs.

Among various types of spinal interneurons related to CPGs, recent molecular and genetic techniques have revealed the existence of several subgroups depending on the functional requirement^10^. In larval zebrafish, for example, different types of spinal neurons are recruited depending on swimming frequency (as reflections of different locomotion modes)^11^,^12^,^13^. Moreover, in mice, focal lesions in specific subgroups of spinal interneurons impair the natural stepping (walking) patterns in a speed-specific manner, even within the same locomotion mode^14^,^15^. Thus, these animal studies using newly developed genetic techniques have demonstrated that there are distinct spinal neuronal groups, which are recruited independently in different locomotor modes and even at different speeds in the same locomotor mode. Thus, the question arises if there are similar spinal neuronal groups that are recruited independently during walking and running in humans. This is the primary research question we aim to answer in a series of studies. In our previous studies using the well-established locomotor adaptation paradigm with a split belt treadmill, we demonstrated that locomotor patterns newly acquired in walking rarely transfer to running^16^,^17^,^18^, suggesting that the neural mechanisms underlying walking and running are not shared, but independent. This result was in line with Vasudevan *et al.*^19^, who showed limited transfer of newly acquired walking patterns across different speeds in the same locomotor adaptation paradigm. These behavioural-based results suggest that similar mode- and/or speed-specific neural mechanisms to those shown in animal studies are shared in humans, which we think are phylogenetically possible. To further explore this possibility in the present study, we applied the electromyographic (EMG) signal decomposition technique (nonnegative matrix factorization; NMF) for extracting motor modules^20^,^21^, i.e., spatially fixed locomotor muscle synergies (Fig. 1). It has been suggested that the locomotor modules are encoded in spatial pattern formation networks, which activate multiple motor neuron pools, in spinal CPGs^22^.

Using decomposition techniques, such as NMF, and principal component analysis (PCA), complex activities of various muscles in human walking have been revealed to be generated by the flexible combination of a small set of modules^23^,^24^,^25^,^26^, i.e., a locomotor module as a functional unit generating functionally relevant patterns of muscle activity^25^,^26^. A recent study on complete-SCI patients has demonstrated that tonic drive to the lumber spinal cord by epidural electrical stimulation can induce coordinated muscle activities in lower limb muscles, which are considered to be generated by a combination of multiple locomotor modules^8^. This result was consistent with recent animal studies using spinalized vertebrates, which demonstrated that extracted modules were organized in the spinal neural circuits^27^,^28^,^29^.

Regarding speed- and mode-dependency of locomotor modules, previous studies in humans have examined those characteristics from the following aspects: 1) mode-dependency (i.e., differences between walking and running); 2) speed-dependency in walking; and 3) speed-dependency in running. As to mode-dependency, a previous study showed changes in muscle weighting of a trunk muscle module^30^. In contrast to the mode-dependency, it is generally accepted that the similar modules are utilized regardless of walking speed^23^,^25^,^30^,^31^. Although the majority of walking modules were certainly shared at a wide range of speeds^23^,^30^,^31^, some differences in the components of the modules were found between different walking speeds in these previous studies^23^,^31^. For example, more than half of the participants used at least one different muscle weighting component of modules between slow walking and self-selected speed walking^31^. Regarding speed-dependency in running, although it has been shown that similar temporal activity patterns of modules were utilized in a slow speed range (5–12 km/h [1.39–3.33 m/s], considered as jogging^30^,^32^,^33^), the differences in the organization of modules (i.e., muscle weightings) are not well established. Thus, it remains unknown whether walking modules are truly unchanged regardless of the speed, and more specifically, whether or not there are faster running modules in humans. Therefore, the present study was designed to reveal whether different locomotor modules are recruited with speed changes in walking and running. In the present study, we extracted locomotor modules from EMGs in walking and running over a wide range of speeds. Although the presence of speed-dependency in human locomotor modules has been debatable, based on the existence of the mode- and/or speed-dependent neural mechanisms of locomotion in animals, we hypothesize that different numbers and types of modules would be extracted from EMGs during walking and running, and also at different speeds in the same locomotor mode. The acceptance of this working hypothesis would provide indirect evidence of mode- and/or speed-dependency of neural networks in human locomotion.

## Methods

### Participants

Eight healthy volunteers (ages 20–31 yr, all male) participated in the study. In addition, to examine faster running speeds, eight well-trained college runners (ages 20–24 yr, all male, experience of intensive running training for 5–11 yr) were also recruited. Each participant gave written informed consent for his participation in the study. The study was in accordance with the Declaration of Helsinki and was approved by the local ethics committee of the National Rehabilitation Center for Persons with Disabilities and Graduate School of Arts and Sciences, The University of Tokyo.

### Experimental setup and design

All participants walked or ran on a treadmill (Bertec, Columbus, OH, USA) with linearly increasing speed (ramp speed condition, acceleration was set to 0.01 m/s^2^). The speed range was adjusted to each group safely but as widely as possible (0.3–4.3 m/s in the non-runner group, 0.3–5.0 m/s in the runner group). These speed-ranges were set as fast as possible within the safe limits ascertained in preliminary experiments. Participants were instructed to either walk or run on the basis of their preference under the given speed. The transition speed from walking to running for all participants ranged from 1.9 to 2.3 m/s. Because the acceleration was very small and the maximum speeds were set as safe per group separately, locomotor movements in all participants were always stable during the experiment.

### Data collection

Three-dimensional ground reaction force (GRF) data were recorded at 1000 Hz from force plates under each belt of the treadmill. Surface EMG activity was recorded from the following 16 muscles on the same side of the trunk and leg as that for kinematic recording: tibialis anterior (TA), gastrocnemius lateralis (LG), gastrocnemius medialis (MG), soleus (SOL), peroneus longus (PL), vastus lateralis (VL), vastus medialis (VM), rectus femoris (RF), biceps femoris (long head, BF), semitendinosus (ST), adductor magnus (AM), tensor fascia latae (TFL), gluteus maximus (GM), gluteus medius (Gmed), rectus abdominis (RA), erector spinae (ES). The EMG activity was recorded with a wireless EMG system (Trigno Wireless System; DELSYS, Boston, MA, USA). The EMG signals were bandpass filtered (20–450 Hz), amplified (with 300 gain preamplifier), and sampled at 1000 Hz.

### Analysis of general gait parameters

GRF data were low-pass filtered with a zero-lag Butterworth filter (5-Hz cut-off). The times of foot-contact and toe-off were determined on a stride-to-stride basis from the vertical component of GRF. Based on the timing of foot-contact and toe-off, stance duration, swing duration, stride duration and double support duration were calculated. Walking and running were defined by the presence and absence of double support time, respectively.

### EMG processing and extraction of locomotor modules

The EMG data were divided into 0.1 m/s bins based on the treadmill speed. As the treadmill speed was continuously increasing and the acceleration was set at 0.01m/s^2^, each speed bin contained 10 seconds’ data. Therefore, there were 40 and 47 speed ranges for non-runners and runners, respectively. In each speed range, the first five to eight consecutive gait cycles (as many as possible in the range, almost eight except at the very slow speed) of the EMG data were used for extraction of locomotor modules. The EMG data were digitally full-wave rectified, smoothed low-pass-filtered with a zero-lag Butterworth filter. The low-pass cut-off frequency influences the smoothing of EMG patterns and thus influences the number of extracted modules^34^. To adequately compare EMG envelopes (i.e., EMG patterns with the same smoothing) of movements performed at different speeds, the low-pass cut-off frequency must be adjusted to each speed condition^34^,^35^. Therefore, the cut-off frequency was adapted to the stride rate in each speed range for each individual to obtain the same pattern smoothing across each speed^36^,^37^ according to the following formula: 10 × stride rate (Hz). The low-pass cut-off frequency ranged between 4.5 to 13.1 Hz. The smoothed EMG data were then time-interpolated over a time base with 200 points for each gait cycle. Then, the EMG amplitude of each muscle was normalized to the maximum value for that muscle over all speeds.

By using nonnegative matrix factorization (NMF), locomotor modules were extracted for each participant from the two types of EMG datasets: each speed range EMG matrix was composed of 16 muscles × (number of stride × 200) variables (i.e., time points) and whole-speed EMG matrices composed by each speed range EMG matrices connected in the direction of time points (i.e., the matrices were composed of 16 muscles× the summation of time points of all 40 [or 47] speed range EMG matrices). NMF has previously been described as a linear decomposition technique^20^,^38^,^39^, according to equation (1):


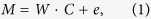


where *M (m* × *t* matrix, where *m* is the number of muscles and *t* is the number of samples, with the spatiotemporal profiles of muscle activity) is a linear combination of muscle weighting components, *W (m* × *n* matrix, where *n* is the number of modules), and temporal pattern components, *C (n* × *t* matrix), and *e* is the residual error matrix. Before the extraction of modules, each muscle data vector was normalized to have unit variance (i.e. standard deviation of each muscle data vector = 1) so as to equally weight the EMG activity across all muscles^31^. This normalization was removed after module extraction to rescale the data to the original scaling and to present small activation of muscle as small. The normalization before module extraction and the removal of normalization is reasonable to reflect small activation of muscles on locomotor modules and provide small but clear patterns^31^,^40^.

The module extraction was iterated 10 times for each possible *n* from 1 to 12, and the variance accounted for (VAF) by the reconstructed EMG (*M*) was calculated at each iteration. The iteration with highest VAF was kept. VAF was defined as 100× uncentered Pearson’s correlation coefficient^41^,^42^. Then, we defined the optimal module number *n* as the number fulfilling the following three criteria. First, *n* was selected as the smallest number of modules that accounted for >90% of VAF^41^. Second, *n* was the smallest number to which adding an additional module did not increase VAF by >5% of VAF^43^. Third, *n* was selected using the “cusp” method, as named by Cheung and colleagues^44^. The cusp method selects the smallest *n* such that the increase of VAF derived from an additional module is lower compared with the increase of 75% of the almost constant VAF slope calculated for random EMG data (i.e., the number beyond which any further increase in the number of extracted synergies yields a VAF increase <75% of that expected from chance). In brief, the VAF curve (a plot of the VAF against the number of modules from 1 to 12) was created from both the original EMG matrix and an unstructured EMG matrix generated by randomly shuffling the original data matrix across muscles and times (i.e., the unstructured EMG dataset has no regularity). Then, *n* was selected as the number at cusp, at which the slope of the original VAF curve falls below 75% of the slope of the shuffled VAF curve. The selected number *n*, beyond which any greater increase in the number of modules contributes to an increase in VAF smaller than 75% of that likely stemming from chance, may be regarded as a reasonable selection for the number of modules.

The number of modules were compared among the whole speed and the six representative speeds in each participant group (nearest speed to 20%, 50%, and 80% of the walking and running speed range in each participant, for the “slow walk”, “moderate walk”, “fast walk”, “slow run”, “moderate run”, and “fast run”, respectively, corresponded to 0.60 ± 0.53, 1.18 ± 0.64, 1.69 ± 0.64, 2.49 ± 0.64, 3.19 ± 0.64, and 3.85 ± 0.53 m/s in non-runners, and 0.70 ± 0.00, 1.28 ± 0.35, 1.79 ± 0.35, 2.74 ± 0.52, 3.60 ± 0.00, and 4.40 ± 0.00 m/s in runners).

### Clustering the modules across participants

To quantify the similarity of modules among participants at each speed, we sorted the extracted modules using hierarchical clustering analysis (Ward’s method, Euclidian distance) of muscle weighting components. The sorting was performed for all participants for each speed condition (the whole speed and the six representative conditions) per group separately. The optimal number of clusters was determined by the gap statistic^45^. Subsequently, the muscle weighting components in each cluster were averaged across subjects. If two components from a participant were put in a same cluster, we retained only one of them (that which had the higher similarity to the cluster average) in that cluster^40^. Although the module having a lower similarity to the cluster average was excluded from the analysis about sorting across participants, these modules were used for the other analyses (EMG reconstruction [described later] and number of modules). Then, correlations were calculated for all pairs of the averaged muscle weighting components between those at the whole speed and for each representative speed. A pair was regarded as the same type of components if it had an *r*-value larger than 0.623^31^.

In addition, corresponding temporal pattern components were also grouped based on the results of the clustering of muscle weighting components. To evaluate activation pattern modulation within a gait cycle, each temporal activation pattern component was averaged across strides. Then, these activation patterns within a gait cycle were averaged across subjects with each cluster. Finally, to quantify the similarity between the averaged temporal pattern components among speeds and between modes, the timing of the main peak was calculated for the muscle weighting components.

### Reconstruction of EMG across all speeds with a set of modules

**Figure 2:**
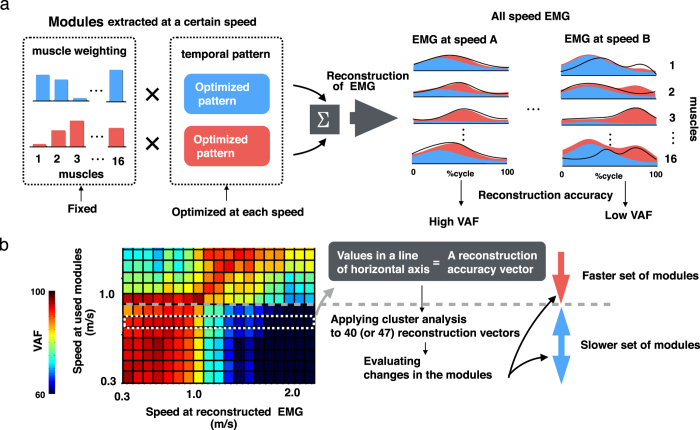
Schematic of the EMG reconstruction method (**a**) and the evaluation of shifts in components of modules based on reconstruction accuracy vectors (**b**). (**a**) EMG reconstruction was accomplished by fixing muscle weighting components (blue and red bars in the left panels) while optimizing the temporal pattern components (blue and red rectangles in the left panels) to reduce the error between the original EMG (black waveforms in the right panels) and reconstructed EMG (areas filled with blue or red in the right panels). Then, variance accounted for (VAF) was calculated between the original and reconstructed EMG. If the components are able to reconstruct the EMG signal with high accuracy, the VAF represents high value (at speed A, left waveforms and filled area). Otherwise, the VAF represents low value (at speed B, waveforms and filled area). This procedure was performed for one set of components at all speeds. That is, a 40- (or 47-) dimensional reconstruction accuracy vector was created for 40 (or 47) speeds. (**b**) The reconstruction matrices (heat maps) were created from the reconstruction accuracy vectors across all speeds. Values in a line along the horizontal axis (surrounded by a white dotted line) represent a reconstruction accuracy vector. The shifts of components of modules (at 0.9 m/s in this example; a grey dashed crossbar between two arrows) were assessed by cluster analysis applied to the overall speeds of reconstruction vectors.

The above-mentioned analyses can evaluate changes in the number of modules and the components of modules (i.e., differences among each module). However, it remains unclear that the quantitative differences between sets of modules among speeds and modes. For example, in our study, the number and types of extracted modules were different between slow walking and fast walking, while only the types of modules were different between fast walking and slow running (described later in the Results section). However, it was difficult to determine which difference is greater using only the evaluation based on the number and component of characteristics. To evaluate differences between sets of motor modules among different conditions, some previous studies^25^,^46^,^47^,^48^ assessed how well a set of modules in one condition reconstructed muscle activities in other conditions. In the present study, we performed EMG reconstruction analysis to evaluate quantitative differences between sets of modules among different locomotor modes and speeds. A detailed schema of this method is shown in Fig. 2. First, we reconstructed EMG activity across all speeds by fixing one set of muscle weighting components extracted from one speed and while optimizing only temporal pattern components to reduce the error between the original and reconstructed EMG for each speed (Fig. 2a). Then, the reconstruction accuracy was evaluated based on the VAF between the original and reconstructed EMG (Fig. 2a, right panels). In this analysis, EMGs had to be reconstructed with a high VAF among the speeds where similar modules were recruited. The procedure was iterated for all speed EMGs for one module set at a certain speed; that is, 40- (or 47-) dimensional reconstruction accuracy vectors were calculated for each module set at 40 (or 47) speeds. Next, reconstruction accuracy matrices (Fig. 2b, heat map) were constructed by vertically combining the reconstruction accuracy vectors as row vectors for each speed (Fig. 2b, an example vector is surrounded by a white dotted line). In the heat map, one line of values on the x-axis indicates the reconstruction accuracy to all speed EMGs of one module set. Subsequently, the matrices were averaged across subjects separately for each participant group. Here, assuming that homogeneous module sets have similar trends in EMG reconstruction accuracy, we evaluated changes of modules among speeds and modes based on the extent to which a component set at a certain speed can reconstruct all speed EMGs. If a specific set of modules is recruited for a certain range of speeds, similar reconstruction accuracies should be observed among the sets of modules extracted in the speed range in any EMGs (i.e., these sets of modules have similar reconstruction accuracy vectors) (Fig. 2b, arrows denote speed ranges of a specific set of modules). Therefore, to quantify the changes in the reconstruction accuracy vectors among speeds, the vectors were grouped by hierarchical clustering (Ward’s method, Euclidian distance). The optimal number of clusters was determined by the gap static method^45^.

### Statistics

Differences in the number of modules were compared among seven speed conditions (whole speed and six representative speeds) per participant group using the non-parametric Kruskal–Wallis one-way analysis of variance (ANOVA) test with the Steel–Dwass post hoc test (nonparametric Tukey’s test). Data are presented as the mean and standard error of the mean (mean ± SE). Statistical significance was accepted at *p* < 0.05.

### Validation of the effect of criterion for the number of modules

In previous studies, criteria to select the module number are broadly classified into two types: “global” and “local” criteria. The “global” criteria is based on the R^2^ (coefficient of determination) or the VAF between the overall reconstructed EMG matrix and the overall original EMG matrix and should be greater than some threshold (e.g., global VAF >90%^43^). The “local” criteria is based on the R^2^ or VAF between reconstructed EMG and original EMG for each individual muscle and needs to be greater than some threshold (e.g., all each muscle VAF >75%^31^). A large number of studies, including the present study, used the “global” criterion^8^,^20^,^24^,^43^,^49^. While many other studies use a combination of “global” and “local” criteria^25^,^31^,^40^. It has been reported that different criteria may lead to different module numbers and components^50^. Therefore, we determined whether the present results would be the same using the criterion combined with global and local VAF. Locomotor modules were extracted from the above-described EMG data based on the same criteria used in a study by Chvatal and colleagues^31^ (global VAF >80% and all each muscle VAF >75%). Using these extracted modules, we performed the above-mentioned analyses (i.e., comparison of the number of modules, and clustering the modules based on weighting components and EMG reconstruction). In addition, it should be noted that there is a possibility that there is less variability in the EMG patterns in slow walking and that all modules used in faster walking are not extracted from slow walking EMG, especially at the same criterion. Thus, we extracted five locomotor modules at all speeds (five modules condition) and tested whether the same modules were extracted among the different modes and speeds. First, five locomotor modules were extracted from the above-described EMG at all speeds. Then, using these extracted modules, the same analyses (i.e., clustering the modules based on weighting components and EMG reconstruction) were performed.

## Results

### Changes in EMG pattern depending on locomotor speed and mode

**Figure 3:**
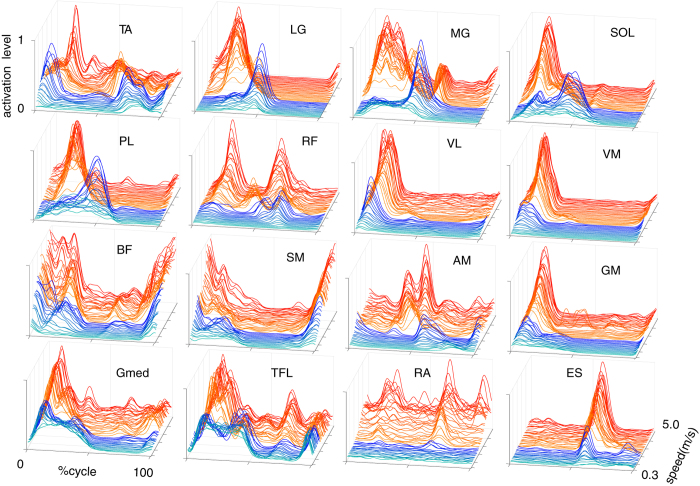
Muscle activation patterns during gait cycles over the course of speeds. Each waveform represents the ensemble average of the first six gait cycles in each speed range in a single participant (runner). The amplitude is normalized to the maximal value for each muscle over all speed ranges in this participant. The waveforms shown in blue and red represent walking and running, respectively.

We recorded electromyographic (EMG) activities from the 16 muscles on one side of the trunk and leg. The EMG activity from a typical participant is shown in Fig. 3. Changes in the EMG patterns depending on locomotor speed and mode were roughly divided into three types based on visual inspection, with the exception of a few others (TA: tibialis anterior, RF: rectus femoris RF, AM: adductor magnus). In the first type, the peak activation level gradually increased with increasing speed regardless of locomotor mode, while the activation timing was nearly constant throughout the majority of proximal leg muscles (GM: gluteus maximus, VL: vastus lateralis, VM: vastus medialis, BF: biceps femoris, ST: semitendinosus) and the trunk muscles (RA: rectus abdominis, ES: elector spinae). In the second type, the activation level increased with increasing speed regardless of locomotor mode, while the activation timing abruptly shifted to an earlier phase at the walk-to-run transition in the triceps surae (SOL: soleus, MG: gastrocnemius medialis, LG: gastrocnemius lateralis) and the peroneus longus (PL). In the third type, clear activity was seen from the slowest walking speed, and the activation durations were gradually shortened with acceleration regardless of locomotor mode in the gluteus medius (Gmed) and the tensor fascia latae (TFL).

### Extracted locomotor modules at different speeds

**Figure 4:**
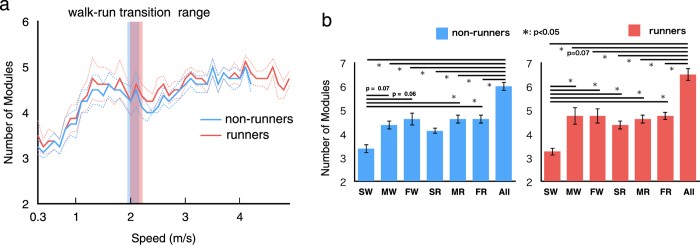
Number of modules over the course of speeds, (**a**) all speed and (**b**) at six representative speeds in non-runners and runners. (**a**) Average number of modules across subjects in all speeds per group (thick lines) and their standard errors (SE, dotted lines) are represented. Translucent areas represent walk-run transition speed range for non-runners (blue) and runners (red). (**b**) The average number of modules at six representative speeds (corresponding to the speeds nearest to 20%, 50%, and 80% over the course of speeds for walking and running in each participant, respectively, for the “slow walk”, “moderate walk”, “fast walk”, “slow run”, “moderate run”, and “fast run”) for non-runners (blue) and runners (red). Ranges of speed of each representative speed in all participants are shown below the condition names. Error bars indicate the SE. SW: slow walking, MW: moderate walking, FW: fast walking, SR: slow running, MR: moderate running, FR: fast running, Whole: whole speed.

**Figure 5:**
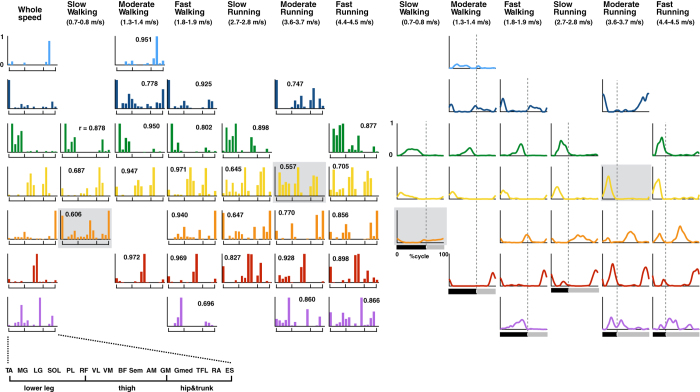
Extracted muscle weighting components and corresponding temporal patterns in whole speed and each representative speed in a representative participant. Each bar height represents the relative level of activation of each muscle within the muscle weighting components. Correlation coefficients between modules for the whole speed and for the representative speed in the same row are shown. The same types of components are represented by the same colour (*r* > 0.623, *p* < 0.01). Modules indicated with grey backgrounds have *r* > 0.497 (*p* < 0.05), but <0.623. An enlarged view of the *x*-axes is shown in the bottom left corner. Temporal pattern components (waveforms) are placed in the corresponding position to their muscle weighting components. The speed for the representative participant is shown below the condition names. The underbars denote stance phase (black) and swing phase (grey) in a gait cycle. The dotted lines denote the transition timing from stance phase to swing phase.

**Figure 6:**
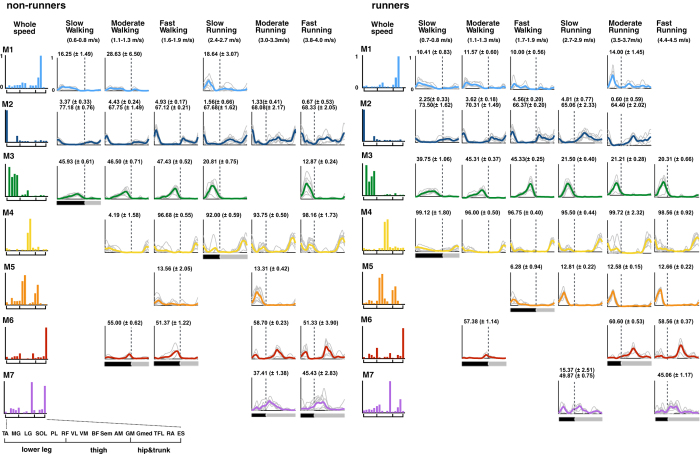
Seven types of muscle weighting components from whole-speed datasets in all participants per group, and shifts in the active components depending on speed and mode and their corresponding temporal pattern components. The averages of each cluster of muscle weighting components from whole-speed datasets are shown in the left column (M1–M7). An enlarged view of the *x*-axes of these components is shown in the bottom left corner. If the components are used in the representative speeds (see Table 1), corresponding temporal pattern components are shown to the right of these components. The peak timings (% cycle) are shown just above each temporal component. Ranges of speed of each representative speed for all participants are shown below the condition names. The underbars denote average stance phase (black) and swing phase (grey) in a gait cycle across subjects. The dotted lines indicate the average transition timing of stance phase to swing phase across subjects.

Using nonnegative matrix factorization (NMF), locomotor modules were extracted in each participant from the two types of EMG datasets: a whole-speed EMG matrix and each speed range EMG matrix. Here, we found that (1) the number of extracted modules changed depending on the locomotor mode and speed (Fig. 4), and (2) different sets of modules, which were extracted from the whole-speed dataset, were recruited depending on locomotor mode and speed (Table 1 and Figs 5 and 6).

Figure 4 shows the number of extracted locomotor modules in each speed range. Approximately four to five modules were extracted over the range of 1.0–5.0 m/s (Fig. 4a), while there were nearly three modules in the slow walking speed range (0.3–1.0 m/s, Fig. 4a). Subsequently, we compared the number of modules among the whole-speed and six representative speeds (i.e., slow, moderate, and fast walk and slow, moderate, and fast run) independently in non-runners and runners. As the ranges of speeds in walking and running were different among participants, the six representative speeds were defined individually for each participant (see Methods). The number of locomotor modules was significantly different among the six representative speeds in non-runners and runners (p < 0.001 for both groups, Kruskal–Wallis one-way ANOVA). Specifically, the number of modules in slow walking was significantly lower than that in moderate run, fast run, and whole speed in non-runners, and the number of modules in slow walking for runners was significantly lower than that in all other conditions (Fig. 4b; *p* = 0.074 [0.030], 0.056 [0.031], 0.11 [0.036], 0.036 [0.021], 0.036 [0.014], and 0.0079 [0.0081] for moderate walk, fast walk, slow run, moderate run, fast run, and whole speed in non-runners [runners], respectively; post hoc Steel–Dwass test). Additionally, the number of modules in the whole-speed dataset was significantly higher than those at all other conditions except in moderate walking in runners (Fig. 4b; *p* = 0.0079 [0.0081], 0.013 [0.074], 0.049 [0.044], 0.0069 [0.0091], 0.018 [0.0091], and 0.018 [0.0081] for slow walk, moderate walk, fast walk, slow run, moderate run, and fast run in non-runners [runners], respectively).

Figure 5 shows a typical example of the extracted components of locomotor modules (muscle weighting components [Fig. 5, bars] and their corresponding temporal activation patterns [Fig. 5, waveforms]) in the six representative speed and whole-speed conditions from a representative participant. In this participant, seven types of modules were extracted in the whole-speed condition, and different sets of the whole-speed modules were extracted for the six speeds.

Next, we examined whether the same types of modules were used among participants and speeds using the hierarchical clustering method. As a result, seven types of modules were extracted from the whole-speed datasets in both groups (Table 1 and Fig. 6). For the six representative speeds, different sets of the whole-speed modules were extracted with changing speed (Table 1 and Fig. 6). Figure 6 shows each type of muscle weighting component of the modules from the whole-speed datasets (bars on the left column) and their corresponding temporal activation patterns for each representative speed (waveforms on the right of the muscle weighting components). As the speed increased, the temporal activation patterns of each module increased in amplitude. Although sorted based on the similarity of muscle weighting components, the peak timings of temporal activation patterns in the same types of modules were also similar among participants (grey waveforms) and speeds (displayed in the same colour). Nevertheless, there were some differences in the peak timings of M3 and M7 depending on mode and speed. In M3, the peak timing was substantially shifted from a peak at around 45% of the cycle during walking to around 20% of the cycle during running. Although M7 has two clear peaks (at initial stance and initial swing) during slow running in runners, the intensity of the first peak diminished during fast running.

### EMG reconstruction

**Figure 7:**
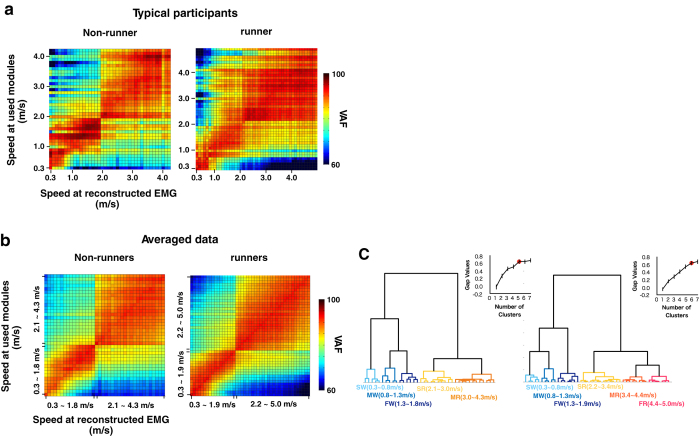
Reconstruction accuracy matrices for representative participants (**a**), averaged data for each participant group (**b**), and results of cluster analysis applied to the averaged data (**c**). (**a,b**) VAF values in a line along the horizontal axis represent a reconstruction accuracy vector for a particular speed. Changes of the values of the vector indicate changes in the components of modules. A colour scale denotes the VAF value. The data in the walk-run transition speed range were excluded when averaging data. (**c**) Dendrograms represent the results of cluster analysis (Ward’s method, Euclidian distance) applied to the averaged data. The line charts show the optimal cluster number based on the gap statistic values. Error bars indicate the SE. The red circles indicate the optimal cluster number. The same clusters are indicated with the same colour. SW: slow walking; MW: moderate walking; FW: fast walking; SR: slow running; MR: moderate running; FR: fast running.

The above-presented results demonstrate that the number and the components of modules changed depending on locomotor speed and mode. To further quantify the speed- and mode-dependent differences among sets of modules, we performed EMG reconstruction^20^,^28^ and cluster analysis. Figure 7a shows heat map representations of the reconstruction accuracy matrices for representative participants in non-runners and runners, and Fig. 7b shows the averaged data across subjects in each group. Because walk-run transition speed was different in each participant, the data at transition speed (non-runner: 1.9–2.1 m/s, runner: 2.0–2.2 m/s) were excluded from these averaged data (Fig. 7b). These heat maps show that the reconstruction accuracy vector of set of modules at each speed (i.e. one line of values on x-axis) changed depending on mode and speed. In the averaged heat maps, the reconstruction vectors were divided into walking and running as two large clusters in both groups (Fig. 7c). The walking cluster was divided into three sub-clusters in both groups, and the running cluster was divided into two and three sub-clusters in non-runners and runners, respectively (Fig. 7c). The structures of the clusters indicate the quantitative differences among sets of locomotor modules. Thus, the results indicate that locomotor modules change greatly between walking and running, and change within the same locomotor mode (i.e., depending on speed).

### Validation of the effect of criterion on the number of modules

Additionally, we tested whether the above-presented results (Figs 4, 5, 6, 7) are observed in different criteria for selection of module number. Supplementary Figs 1–3 show the number of modules, extracted module types, and reconstruction accuracy vectors in the case of criteria combined with global and local VAF. As this criterion was more severe compared with those for the above results, on average across subjects, 6 to 11 modules were extracted in all speeds (Supplementary Fig. 1a). When comparing the six representative speeds and whole speed conditions, the number of modules was different among speeds (Supplementary Fig. 1b). The types of extracted locomotor modules were changed among six representative speeds (Supplementary Fig. 2). In addition, the reconstruction accuracy vectors were divided into three clusters in non-runners (slow and fast walking and running clusters), and four clusters in runners (slow and fast walking clusters and slow and fast running clusters) (Supplementary Fig. 3).

Supplementary Figs 4 and 5 show the extracted module types and reconstruction accuracy vectors in the case of five locomotor modules at all speeds. Seven types of modules similar to the above results (Table 1 and Fig. 6) were extracted, and different types of modules were used among six representative speeds except between moderate and fast running in non-runners, and between slow and fast running in runners (Supplementary Fig. 4). The reconstruction accuracy vectors were divided into four clusters in both groups (slow and fast walking clusters and slow and fast running clusters, Supplementary Fig. 5).

Together, the results obtained under the two different conditions indicate that, although some results were different to the results presented in the above section (Figs 4, 5, 6, 7), the main findings were the same regardless of the module number selection method: 1) different locomotor modules were used depending on mode and speed; and 2) reconstruction accuracy vectors differed with changes in mode and speed.

## Discussion

In the present study, assuming that human locomotor networks have speed- and mode-dependency as demonstrated in other vertebrates, we tested the following hypothesis using the motor module extraction methods: recruitment patterns of locomotor modules would shift depending on locomotor mode and/or speed. The main results were: (1) the number of modules changed depending on the mode and speed; (2) different types of modules were extracted among all six representative speeds; and (3) the reconstruction vectors were divided into two large clusters of walking and running, and both the walking and running clusters were divided into two or three sub-clusters. The structures of the clusters indicate the quantitative differences among sets of locomotor modules. In summary, these results suggest that different locomotor modules are probably recruited depending on locomotor speed and mode, which confirms our working hypotheses. The present results provide indirect evidence for mode- and speed-dependency in the neural networks underlying human locomotion.

In the present study, based on mathematically extracted motor modules, we tested speed- and mode-dependency in neural networks for human locomotion. The validity of the procedure is strongly supported by direct evidence that spinal neural networks encode locomotor modules, which has been obtained from spinalized animals^21^,^27^,^29^,^51^ and SCI patients^8^. In studies applying factorization algorithms to spinalized animals, it has been suggested that the locomotor modules are encoded in the spinal neural networks^21^,^27^,^29^,^51^. For example, using a spike-triggered averaging method, Hart and Giszter^29^ revealed direct relationships between the spiking of the spinal interneurons and the output of modules extracted by factorization algorithms. Likewise, it has been suggested that locomotor modules exist in spinal cord using cutaneous^51^, electrical^21^ and neurochemical^27^ stimulation combined with factorization algorithms. Similarly, a study that applied epidural electrical stimulation to the lumbar spinal cord of complete-SCI patients combined with factorization algorithms showed that rhythmic muscle activities of the lower limb were generated by a combination of multiple modules^8^. Based on these previous studies, mathematically extracted locomotor modules most likely represent spinal neural networks, which underlie coordinated patterns of muscle activity. Therefore, speed- and mode-dependent changes in extracted modules found in the present study strongly suggest that active spinal locomotor networks shift in a speed- and mode-dependent manner.

Assuming that the locomotor modules are encoded in the spinal cord, the speed- and mode-dependent recruitment of locomotor networks observed in the present study is supported by evidence from some animal studies. Recently, studies using genetic and electrophysiological methods have revealed that spinal interneurons are important components of CPGs, and each interneuron play particular roles in controlling locomotion^52^,^53^. Some vertebrate studies have reported that specific sets of spinal interneurons are recruited depending on locomotion speed and/or mode^11^,^13^,^15^,^54^. For walking in mice, it was demonstrated that two subtypes of interneurons regulating left-right limb alternation could be identified, and differences were found in their contribution to left-right alternation depending on walking speed^15^. Namely, although slow walking and fast walking have been defined as the same locomotor mode, the neural mechanisms underlying slower and faster walking are clearly different in mice. In addition, the larval zebrafish exhibits two different locomotor modes depending on swimming frequency^1^, and different classes of spinal interneurons are recruited depending on swimming frequencies corresponding to different locomotor modes^11^,^12^,^13^. Together, these previous results show that appropriate interneuronal locomotor modules are selected and combined within hard-wired modules depending on speed and mode based on the on/off switching of active modules. In the present study, active modules at certain speeds consisted of a combination of parts of modules extracted from whole-speed EMG datasets (Table 1 and Fig. 6). The results are similar to the characteristics of interneuronal modules in other vertebrates as regards to the recruitment of locomotor modules for speed and mode control. Although it is not clear whether such mode- and speed-dependent recruitment mechanisms of spinal interneurons can be extended to humans, the characteristics of core components of CPGs, which have been mainly derived from a set of embryonic interneurons, are remarkably conserved across different species, even between fish and rodents^52^. In fact, there is substantial evidence that CPG-like neural networks are preserved in humans, and they have been mostly observed in patients with chronic SCI^5^,^6^,^8^. Also, as in quadrupeds, long projecting propriospinal neurons connect the cervical and lumbar enlargements in humans^55^. Additionally, during gait, coordination and patterns of reflex mediated at the spinal cord are quite similar in humans^56^ and cats^57^. Based on these observations, the coordination of human gait seems to be controlled by the spinal neural mechanisms in much the same way as gait in other vertebrates. Similarly, if the mode- and speed-dependent recruitment mechanisms of spinal interneurons for locomotion are phylogenetically preserved in humans, this would explain the speed- and mode-dependent specificity of locomotor modules found in the present study.

Some previous studies on human locomotion compared locomotor modules among modes and speeds^23^,^25^,^30^,^31^. Between walking and running, a study of them changes in the muscle weighting component of trunk extensors/adductors (correlation of the modules between walking and running: r = 0.38, *p* > 0.05)^30^. Conversely, regarding speed-dependency, some prior studies have argued that similar locomotor modules have been extracted while walking in various speeds, in contradiction to our results^23^,^25^,^31^. Nevertheless, a study^31^ extracting locomotor modules from self-selected (1.2–1.5 m/s) and slow walking speeds (0.6 m/s) reported that one locomotor module was only used at one walking speed in six out of nine participants. Thus, minor differences in locomotor modules that depended on speed and mode were found in these previous studies.

Methodological differences may have caused the differences in the results between the previous studies and ours. It has been proposed that the extracted modules are affected by the number of muscles, and which muscles are recorded^50^. Some of the previous studies did not record Gmed^23^ or TFL^25^ activities, which are major muscles in a module mainly extracted in slow and moderate walking (Fig. 6, M1). Therefore, it might not have been possible to sufficiently demonstrate the differences between slow (or moderate) walking and fast walking modules in these studies. In addition, the criteria used to determine the number of modules may also be a factor in the differing results. Indeed, it has been reported that different criteria lead to different module numbers and structures^50^. Therefore, differences in the criteria adopted between the previous and present studies might lead to the difference in the results related to speed dependency. Nevertheless, our findings were consistently observed under the three different conditions for selecting the number of modules global VAF criterion Figs 3, 4, 5, 6, 7, criterion combining global and local VAF (Supplementary Figs 1–3) and the five modules condition (Supplementary Figs 4 and 5). Based on the results, our findings are most likely not methodology-dependent.

Regarding the speed-dependency of locomotor modules, the present method that uses EMG reconstruction with clustering provides additional information about the quantitative differences between the sets of modules among speeds and modes. In some previous studies^23^,^31^, the differences among speeds at each module level (i.e., the number of modules and the characteristics of components) were the main targets of analysis. Using these parameters, it has been difficult to compare the quantitative differences among sets of modules, particularly in cases where two sets have a different number of modules. In the present study, using EMG reconstruction methods, one set of modules at a speed was evaluated by reconstruction accuracy to muscle activities of wide speed locomotion. The reconstruction accuracy vectors were divided into walking and running as two large clusters, then the walking and running clusters was divided into two or three sub-clusters (Fig. 7). The structure of the clusters indicates quantitative differences among sets of modules, thus the results mean that the locomotor modules greatly differed between walking and running, and were also different among the same locomotor mode because they depended on speed. Conversely, in many previous studies, the similarity of the modules has been evaluated based on the correlation coefficient between each module. If two different behaviours use multiple similar modules just barely exceeding the criteria of similarity, the modules of the two behaviours would have been different as a whole set of modules. By using the present EMG reconstruction method combined with clustering, the qualitative difference of component sets can be identified. Therefore, if our method had been used in previous studies^23^,^31^, the walking modules might have been divided into different clusters depending on speed.

Regarding the speed-dependency of locomotor modules, a previous study^25^ performed a similar EMG reconstruction method to ours. The study reconstructed the walking EMGs over a wide range of speeds (0.3–1.8 m/s) with a high accuracy, using only one module set extracted from a self-selected speed (1.25 m/s on average). They assumed that “if the reconstruction of Y by using X is conducted with a high accuracy, then that generally means that X and Y recruit the same modules”, and therefore they concluded that the same locomotor modules are recruited in various walking speeds. In our EMG reconstruction method, we did not assume the same principle because it is inappropriate for our results. For example, as the moderate speed walking modules contained almost all of the modules in slow walking (Fig. 6 and Table 1), the moderate walking speed modules could reconstruct EMGs at slow walking with a high accuracy (Fig. 7b). On the contrary, as the slow walking modules contained only some of the modules used in moderate speed walking (Fig. 6 and Table 1), these modules could not well reconstruct EMGs in moderate speed walking (Fig. 7b). Therefore, we evaluated the reconstruction vectors from another viewpoint. Assuming that homogeneous module sets have similar trends in EMG reconstruction accuracy, we evaluated the changes of modules among speeds and modes based on the extent to which a module set at a certain speed can reconstruct all speed EMGs by using hierarchical clustering. This method enabled evaluating important information about the similarity of modules among speeds and modes in the reconstruction vectors. For example, the sets of modules in slow walking (0.3–0.8 m/s) reconstructed running EMGs with only low accuracies (about 60% VAF) (Fig. 7b). On the other hand, those of fast walking (1.3–1.8 m/s) reconstructed running EMGs with fairly high accuracies (about 80% VAF) (Fig. 7b). This means that modules in fast walking contained those required in running, and suggests that there are clear differences between slow walking modules and fast walking modules. In this method, although the clusters of reconstruction vectors were divided mathematically (Fig. 7c), each set of modules in each representative speed, which corresponded to speed range of each cluster, had different sets of locomotor modules (Fig. 6). For example, fast walking modules contained a large portion of slow walking modules (2/3 modules in non-runners, 4/4 modules in runners, Table 1 and Fig. 6), while slow walking modules did not contain many fast ones (2/5 modules in non-runners, 4/5 modules in runners, Table 1 and Fig. 6). It has been shown that each type of motor module has a particular biomechanical function, and it has been suggested that the modules act as basic neural control elements^20^,^21^,^26^. Thus, as discussed in previous studies at each muscle level^58^,^59^, the results suggest that the different modules were recruited among different walking speeds to presumably meet additional functional demands with a speed increase (e.g., deceleration of the leg in late swing [M4] and large loading response in initial stance [M5]). Therefore, it is considered that the differences among the clusters of reconstruction vectors have important physiological relevance to the speed-dependent recruitment of locomotor modules.

Although the present study mainly focused on the muscle weightings of modules (i.e., spatially fixed muscle synergies), Ivanenko and colleagues have extensively studied the timing patterns of locomotor modules with regard to speed dependency and mode dependency^23^,^30^. Their studies showed that the peak timing shift of the locomotor module activities depended on the speed and mode. One of their studies indicated a peak timing shift of a temporal pattern component of ankle extensors between walking and running (~45% of the cycle during walking, 20–30% of the cycle during running)^30^. Concerning speed dependency, another study showed a main activity peak of a type of temporal component mainly controlling BF and ST shifted from the late swing phase at 2, 3, and 5 km/h (0.56, 0.83, and 1.39 m/s) to the early stance phase at 1 km/h (0.28 m/s)^23^. In addition, the activity peaks of all types of temporal components shifted earlier about 9% on average, depending on acceleration from 1 to 5 km/h (0.28–1.39 m/s)^23^. Similarly, in our study, some changes in the timing patterns of the modules were found (Fig. 6). The peak timing of M3 was substantially shifted from a peak at around 45% of the cycle during walking to around 20% of the cycle during running. Additionally, M7 had two clear peaks (at initial stance and initial swing) during slow running in runners; however, the intensity of the first peak diminished during fast running. In addition, as the speed increased, the temporal activation patterns of each module increased in amplitude (Fig. 6). Thus, on the basis of the prior studies and present results, the temporal activity patterns of each type of spatially fixed locomotor modules are adjusted depending on speeds and modes. These results suggest that the temporal and spatial aspects of locomotor pattern generation are separately and hierarchically organized, and the observation is consistent with a simulation study of locomotor CPGs that demonstrate that temporal rhythm generators in the spinal cord control the networks of spatial pattern formation^22^.

Between non-runners and runners, different sets of modules were extracted at all representative speeds (Fig. 6 and Table 1). In addition, another faster running sub-cluster of EMG reconstruction accuracy vectors was observed in runners compared with non-runners (Fig. 7). Although locomotor networks have inherent neural mechanisms^24^, plastic changes occur in the networks with locomotor training^60^,^61^. The present participants in the runners group have undergone intensive locomotive training for at least 5 years. Therefore, the possibility remains that long-term training causes plastic changes in locomotor modules. In a study targeting chronic stroke patients, it has been demonstrated that alterations of modules in the stroke-affected arm reflect a fractionation of the unaffected-arm modules^62^. It has also been demonstrated that the use of existing modules is an efficient way to adapt to perturbations in a study of upper-limb movement^47^. Likewise, in the present study, specific sets of modules in runners were constructed using the modules extracted at other speeds or from non-runners (Fig. 6 and Table 1). It is possible that the acquisition of novel locomotor movement following long-term training is achieved via the reorganization of locomotor networks consisting of existing locomotor modules. However, one possible limitation is that the two groups were compared under different speed ranges. As the maximum speed for runners was set to faster (5.0 m/s) compared with non-runners (4.3 m/s) to examine faster speed running, the representative fast running speed differed greatly between non-runners and runners. However, the representative slow walking, fast walking, and slow running were approximately the same speed between the two groups. Therefore, it seems valid to conclude that extracted modules in the two groups were different at these three representative speeds.

In conclusion, our results revealed that different numbers and types of modules are utilized depending on the speed and the mode. This result strongly suggests the existence of mode- and speed-specificity in neural networks for human locomotion.

## Additional Information

**How to cite this article**: Yokoyama, H. *et al.* Distinct sets of locomotor modules control the speed and modes of human locomotion. *Sci. Rep.* **6**, 36275; doi: 10.1038/srep36275 (2016).

**Publisher’s note:** Springer Nature remains neutral with regard to jurisdictional claims in published maps and institutional affiliations.

## Supplementary Material

Supplementary Information

## Figures and Tables

**Figure 1:**
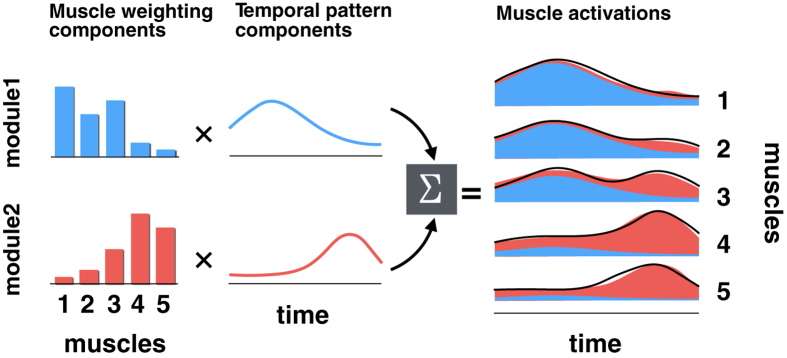
Schematic model of EMG reconstruction by the sum of muscle activation generated by different modules (five muscles and two modules, for example). The output of each module (areas filled with blue or red in the right panel) is explained by the product of the muscle weighting component (bars in the left panels; showing activation level of each muscle) and the temporal pattern components (lines in the middle panels). Consequently, the total muscle activation (black lines in the right panel) is explained by the sum of muscle activations generated by each module (filled areas).

**Table 1:** Characteristics of modules and number of participants within the clusters of modules.

	Timing	Major Muscles	Number of participants within clusters						
			(non-runners/runners)						
			Whole	SW	MW	FW	SR	MR	FR
M1	Early stance	TFL, Gmed	7/8	5/6	4/7	−/4	7/−	−/4	−/−
M2	Initial stance/Mid swing	TA	8/8	8/8	8/8	8/8	8/8	6/5	6/−
M3	Mid-late stance	MG, LG, SOL, PL	8/8	8/8	8/8	8/8	8/8	−/7	8/8
M4	Late swing-Early stance	BF, SM	8/7	−/4	8/7	4/6	7/7	8/7	8/8
M5	Early stance	RF, VL, VM, GM, Gmed, TFL	4/6	−/−	−/−	8/7	−/8	8/6	−/6
M6	Late stance/Mid swing	ES, RF	5/8	−/−	4/4	8/−	−/−	5/5	6/8
M7	Initial swing	AM, RA, ES	4/6	−/−	−/−	−/−	−/4	6/−	7/8

Whole: whole speed, SW: slow walking, MW: moderate walking, FW: fast walking,

SR: slow running, MR: moderate running, FR: fast running.
