# Supplementary material for: Distinct sets of locomotor modules control the speed and modes of human locomotion
Source: Sci Rep. 2016 Nov 2;6:36275. doi: 10.1038/srep36275 (PMC5090253; doi:10.1038/srep36275)
Supplement: Supplementary Information [file srep36275-s1.pdf]

Supplementary Information

Title

Distinct sets of locomotor modules control the speed and modes of human locomotion

Authors

Hikaru Yokoyama,[1,3] Tetsuya Ogawa,[1] Noritaka Kawashima,[2] Masahiro Shinya,[1] Kimitaka Nakazawa[1]

[1] Department of Life Sciences, Graduate School of Arts and Sciences, The University of Tokyo, 3-8-1 Komaba, Meguro, Tokyo 153-8902, Japan

[2] Department of Rehabilitation for the Movement Functions, Research Institute of National Rehabilitation Center for the Disabled, 4-1 Namiki, Tokorozawa, Saitama 359-8555, Japan

[3] Research Fellow of the Japan Society for the Promotion of Science, Tokyo, Japan

Corresponding author:

Kimitaka Nakazawa

Department of Life Sciences, Graduate School of Arts and Sciences, The University of Tokyo, 3-8-1 Komaba, Meguro, Tokyo, Japan

Tel.: +81-3-5454-6869

E-mail address: nakazawa@idaten.c.u-tokyo.ac.jp

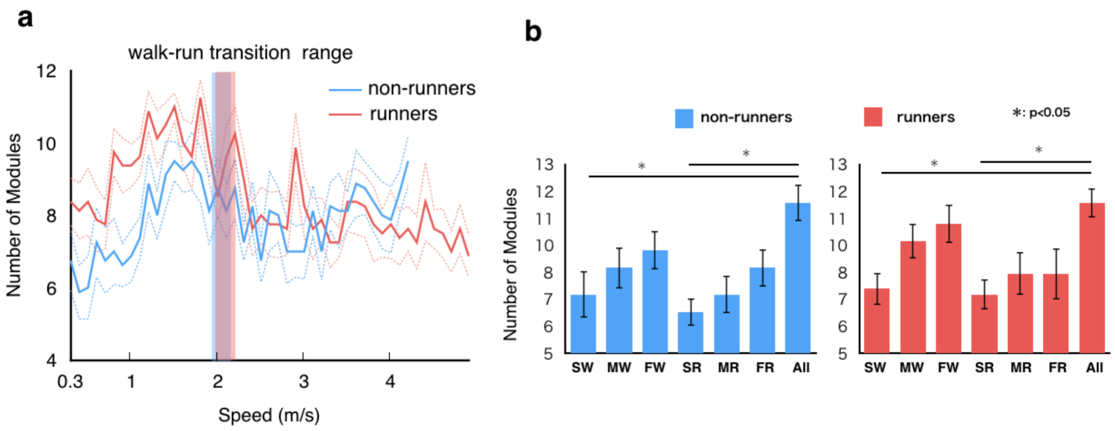

Supplementary Figure 1. Number of modules determined by Chvatal's criterion.

(a) Average number of modules across all speeds in each group (thick lines) and their standard errors (SE, dotted lines) are represented. Translucent areas represent walk-run transition speed range for non-runners (blue) and runners (red). (b) The average number of modules at six representative speeds (corresponding to the speeds nearest to 20%, 50% and 80% over the course of speeds for walking and running in each participant, respectively, for the "slow walk", "moderate walk", "fast walk", "slow run", "moderate run" and "fast run") for non-runners (blue) and runners (red). The number of modules was significantly different among the six representative speeds in non-runners and runners (p = 0.017 and 0.0045 for non-runners and runners, respectively; Kruskal–Wallis one-way ANOVA). In both non-runners (left) and runners (right), compared among the six representative speeds and whole speed condition, the number of modules in slow walking and slow running was significantly lower than that of the whole-speed condition (p = 0.047 [0.039] and 0.034 [0.040] for slow walking and slow running in non-runners [runners], respectively; post hoc Steel–Dwass test). The range of speeds for each representative speed in all participants are shown below the condition names. Error bars indicate the SE. SW: slow walking, MW: moderate walking, FW: fast walking, SR: slow running, MR: moderate running, FR: fast running, Whole: whole speed.

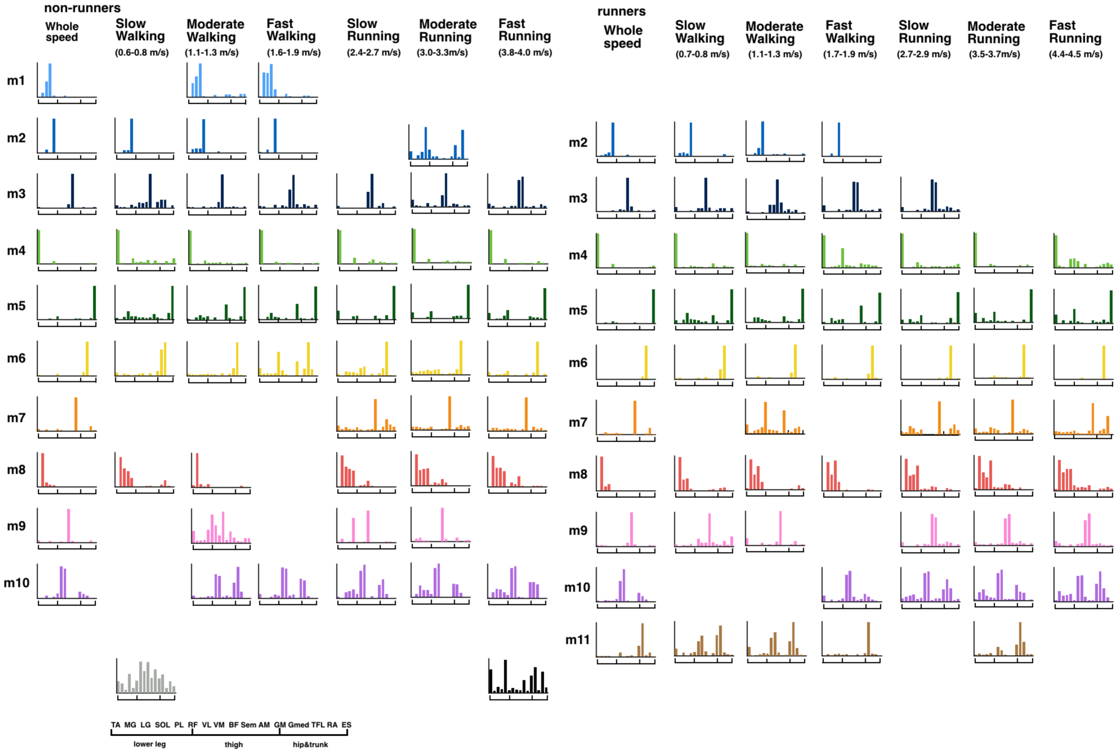

Supplementary Figure 2. Extracted weighting components per group, and change in the extracted components depending on speed and mode in the case of the number of modules determined by Chvatal's criterion.

The average of each cluster of muscle weighting components from whole-speed datasets are shown in the left column (m1–m10 in non-runners, m2–m11 in runners). An enlarged view of the *x*-axes of these components is shown in the bottom left corner. The range of speeds for each representative speed for all participants are shown below the condition names.

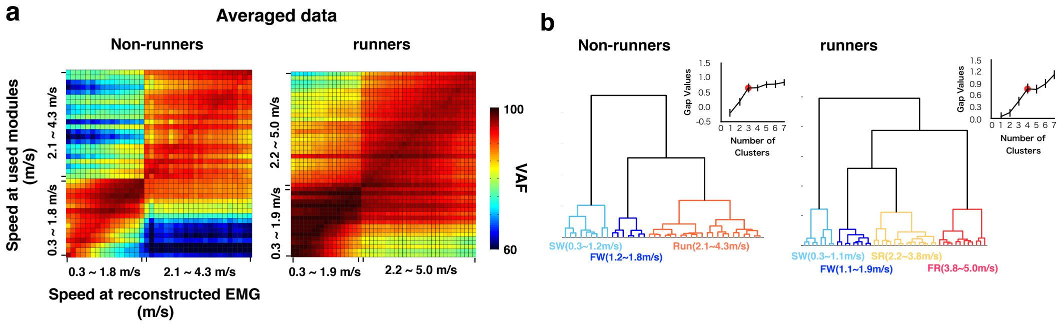

Supplementary Figure 3. Reconstruction accuracy matrices of each component for averaged data for each participant group (a), and results of cluster analysis applied to the averaged data (b) in the case of the number of modules determined by Chvatal's criterion.

(a) VAF values in a line along the horizontal axis represent a reconstruction accuracy vector for a particular speed. Changes in the values of the vector indicate changes in the components of modules. A colour scale denotes the VAF value. Data in the walk-run transition speed range were excluded when averaging data. (b) Dendrograms represent the results of cluster analysis (Ward's method, Euclidian distance) applied to the averaged data. The line charts show the optimal cluster number based on the gap statistic values. Error bars indicate the SE. The red circles indicate the optimal cluster number. The same clusters are indicated with the same colour. SW: slow walking; FW: fast walking; SR: slow running; FR: fast running.

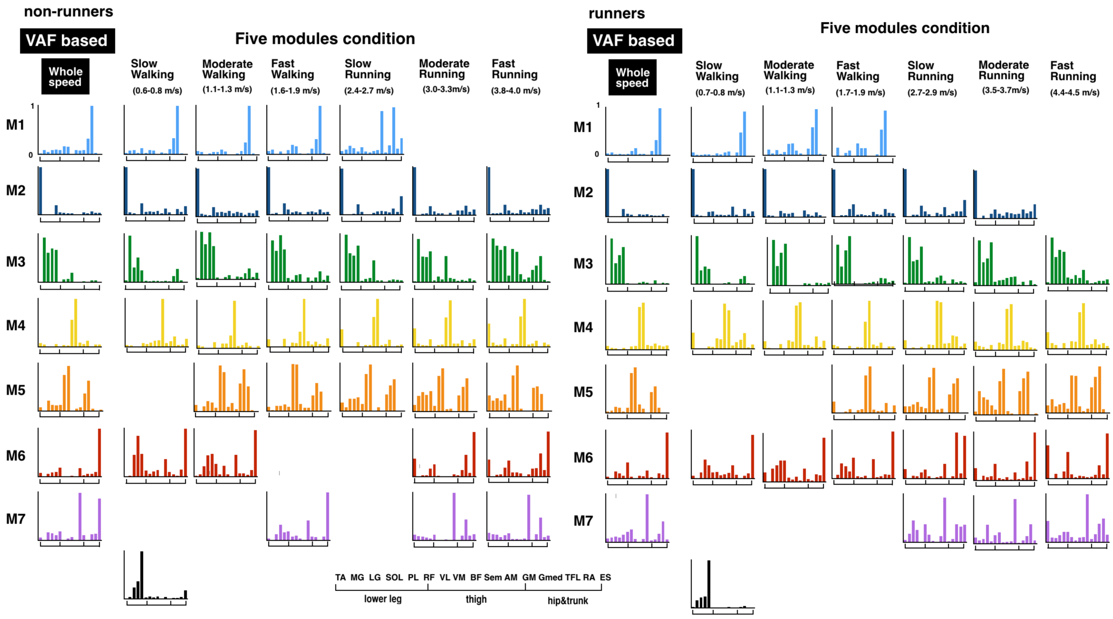

Supplementary Figure 4. Changes in the extracted modules depending on speed and mode in cases where the number of modules was assumed to be five (five modules condition).

The modules in the left column (M1–M7) are the different types of modules extracted from whole speed data using VAF based criterion (i.e. same modules presented in Fig. 6). Based on these modules, modules under the five modules condition at six representative speeds were sorted in the same way as Fig. 6. The range of speeds for each representative speed for all participants are shown below the condition names. An enlarged view of the *x*-axes of these components is shown in the bottom left.

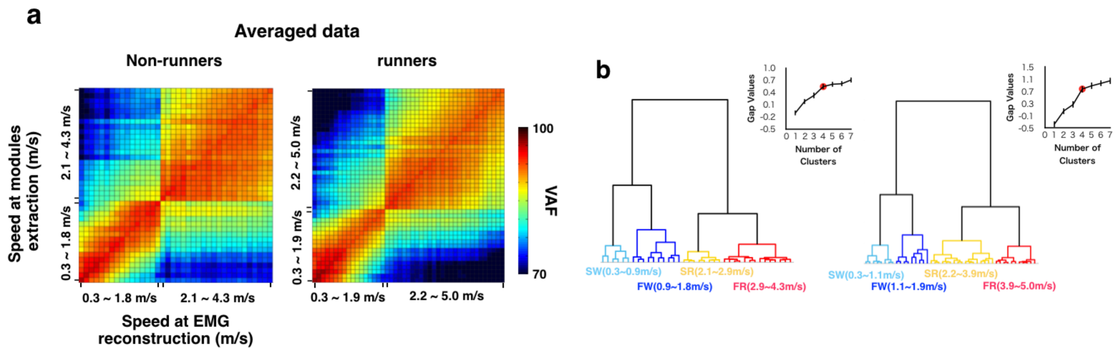

Supplementary Figure 5. Reconstruction accuracy matrices of each component for averaged data for each participant group (a), and results of cluster analysis applied to the averaged data (b) in cases where the number of modules was assumed to be five.

(a) VAF values in a line along the horizontal axis represent a reconstruction accuracy vector for a particular speed. Changes in the values of the vector indicate changes in the components of modules. A colour scale denotes the VAF value. The data in the walk-run transition speed range were excluded when averaging data. (b) Dendrograms represent the results of cluster analysis (Ward's method, Euclidian distance) applied to the averaged data. The line charts show the optimal cluster number based on the gap statistic values. Error bars indicate the SE. The red circles indicate the optimal cluster number. The same clusters are indicated with the same colour. SW: slow walking; FW: fast walking; SR: slow running; FR: fast running.
